# Mandatory chromosomal segment balance in aneuploid tumor cells

**DOI:** 10.1186/1471-2407-7-21

**Published:** 2007-01-26

**Authors:** Maria Kost-Alimova, Eva Darai-Ramqvist, Wing Lung Yau, Agneta Sandlund, Ludmila Fedorova, Ying Yang, Irina Kholodnyuk, Yue Cheng, Maria Li Lung, Eric Stanbridge, George Klein, Stefan Imreh

**Affiliations:** 1 Department of Microbiology, Tumor and Cell Biology (MTC), Karolinska Institute, Box 280, 171 77 Stockholm, Sweden; 2 Department of Biology, Center for Cancer Research, Hong Kong University of Science and Technology, Clear Water Bay, Kowloon, Hong Kong (Special Administrative Region), China; 3 Department of Microbiology and Molecular Genetics, University of California, Irvine, California, USA

## Abstract

**Background:**

Euploid chromosome balance is vitally important for normal development, but is profoundly changed in many tumors. Is each tumor dependent on its own structurally and numerically changed chromosome complement that has evolved during its development and progression?

We have previously shown that normal chromosome 3 transfer into the KH39 renal cell carcinoma line and into the Hone1 nasopharyngeal carcinoma line inhibited their tumorigenicity. The aim of the present study was to distinguish between a qualitative and a quantitative model of this suppression. According to the former, a damaged or deleted tumor suppressor gene would be restored by the transfer of a normal chromosome. If so, suppression would be released only when the corresponding sequences of the exogenous normal chromosome are lost or inactivated. According to the alternative quantitative model, the tumor cell would not tolerate an increased dosage of the relevant gene or segment. If so, either a normal cell derived, or, a tumor derived endogenous segment could be lost.

**Methods:**

Fluorescence in Situ Hybridization based methods, as well as analysis of polymorphic microsatellite markers were used to follow chromosome 3 constitution changes in monochromosomal hybrids.

**Results:**

In both tumor lines with introduced supernumerary chromosomes 3, the copy number of 3p21 or the entire 3p tended to fall back to the original level during both *in vitro *and *in vivo *growth. An exogenous, normal cell derived, or an endogenous, tumor derived, chromosome segment was lost with similar probability. Identification of the lost versus retained segments showed that the intolerance for increased copy number was particularly strong for 3p14-p21, and weaker for other 3p regions. Gains in copy number were, on the other hand, well tolerated in the long arm and particularly the 3q26-q27 region.

**Conclusion:**

The inability of the cell to tolerate an experimentally imposed gain in 3p14-p21 in contrast to the well tolerated gain in 3q26-q27 is consistent with the fact that the former is often deleted in human tumors, whereas the latter is frequently amplified. The findings emphasize the importance of even minor changes in copy number in seemingly unbalanced aneuploid tumors.

## Background

Euploid chromosome balance is vitally important for normal development. Changes in autosomal copy number are lethal or lead to serious developmental anomalies. Normal human cells tend to maintain a diploid chromosome complement in culture. Cancer cells are aneuploid, as a rule. This has been interpreted in different ways during the history of cancer cytogenetics. T. Boveri has proposed in 1914 that all tumors are due to chromosomal changes. Extensive examination of cancer chromosomes in the first half of the 20^th ^century by the crude cytogenetical methods that were available gave a picture of total disorder. This has led to the view that aneuploidy is a consequence rather than a cause of cancer development. More discriminating cytogenetic and molecular techniques detected an increasing number of specific tumor-associated chromosomal changes during subsequent years, but the broadly variable background was still regarded as non-specific "noise". More recent technologies, particularly Spectral Karyotyping (SKY) and Comparative Genomic Hybridization (CGH) identified an increasing number of repeatedly occurring, tumor type specific chromosomal segment changes that were not apparent on ordinary cytogenetic examination. It may be therefore questioned whether any recurrent change can be dismissed as noise. Given the dynamics of tumor cell growth and the plasticity of the karyotype, it is conceivable that all persisting aberrations may have a selective value.

In the present study we have asked whether the copy number of a given chromosomal segment may be critically important for the growth of an aneuploid tumor, so that no quantitative changes would be tolerated. Are some chromosome segments prone to losses and others to gains in a functionally relatable fashion?

It is known that the short arm of chromosome 3 is frequently deleted, while 3q is often amplified in variety of human tumors [1,2]. In order to examine whether the equilibrium of a certain chromosome 3 (chr3) segment copy number is critical for an aneuploid tumor, we have introduced one or two supernumerary chrs3 by microcell mediated chromosome transfer into two different carcinoma lines, the nonpapillary renal cell carcinoma (RCC) derived KH39 and the nasopharyngeal carcinoma (NPC) derived Hone1. Both tumor types are characterized by frequent chr3 changes that lead to 3p losses and 3q gains [3-5]. KH39 is hyper-diploid with few chromosomal anomalies, it carries two morphologically normal chrs3 [6]. Hone1 is hypo-tetraploid with a large number of segmental imbalances. Chr3 is rearranged and participates in 8 markers [7]. Deletions, duplications and imbalanced translocations resulted in very complex picture of segmental imbalances along the chr3 in Hone1 (our unpublished data). Importantly for us, 3p was involved in losses, while 3q in gains.

**Figure 1:**
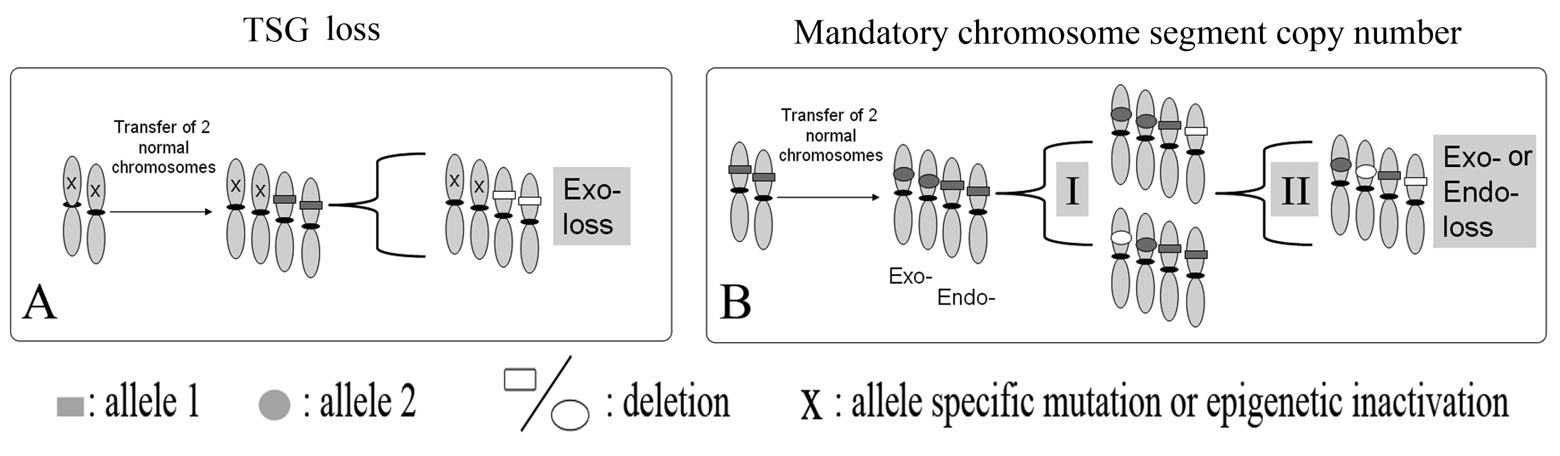
**Chromosomal changes, expected on the basis of the "TSG loss" and the "mandatory chromosomal segment copy number" models, respectively**. **A. **The putative TSG is inactivated in the recipient tumor cell. Following the transfer of normal chromosomes, only the loss of exogenous TSG copies would provide the cell with selective growth advantage. **B. **The recipient tumor cell must maintain equilibrium with regard to the total copy number of certain chromosomal segments. Transfer of supernumerary chromosomes is followed by the restoration of the original balance by a multi-phase expansion of clones, losing first one, then the other extra-copy of the critical regions, irrespectively of their endo- and exogenous origin.

Using these cell lines we have previously shown that the introduction of a normal chr3 inhibited tumor growth [7,8]. Analysis of the tumors derived from the KH39 background microcell hybrids (MCHs) identified regular losses affecting the 3p14.3-p21.3 region [8]. The deleted regions corresponded to or overlapped with the Common Eliminated Region 1 (CER1) and the Frequently Eliminated Region (FER), previously defined through their elimination from human chr3 mouse fibrosarcoma MCHs during *in vivo *growth [9-11]. The 3p21.3 region was also shown to be important for chr3 transfer mediated tumor suppression in Hone1 based MCHs [7]. This was taken to suggest that 3p21 harbors tumor suppressor gene(s) (TSG) that are deleted or inactivated in the tumors. Normal chr3 transfer would inhibit tumor growth by restoring the missing function. If so, the loss of relevant TSGs from the normal exogenous chr3 could release the suppression [12]. Our findings did not confirm this expectation in KH39 derived MCHs [8]. Regular loss of supernumerary 3p copies from the MCHs after tumor growth was not necessarily accompanied by loss of heterozygosity (LOH), expected if the "TSG loss" model is valid (figure 1A). The alternative "mandatory chromosomal segment copy number" hypothesis (figure 1B) implies that the tumor karyotype is finely tuned with regard to the copy number at the critical region and tolerates no experimental additions.

The purpose of the present study was to explore the following questions (figure 1):

1. If MCHs carried more than 1 exogenous chr3 copies, would loss of each supernumerary copy provide the cell with growth advantage and permit clonal expansion?

2. Would the lost segments necessarily originate from the transferred normal chr3 or could they also originate from the endogenous, tumor derived sequences?

3. Is the resistance to copy number change enforced with equal stringency for different chr3 segments?

The results of our study support the hypothesis of "mandatory chromosomal segment copy number". Comparing losses of different chr3 segments, we found that the stringency of copy number maintenance was particularly strong for 3p14-p21. It is weaker for other 3p regions. In contrast, the increase in copy number was well tolerated by 3q26-q27.

## Methods

### 1. Cell lines

MCH A9Hytk3 and MCH 903.1 containing a single cytogenetically intact human chr3 on mouse fibrosarcoma A9 background [12,13], were used as chr3 donors for microcell mediated chromosome transfer as described [14].

Hygromycin resistance gene tagged chr3 from MCH A9Hytk3 was transferred into nonpapillary (clear cell) RCC cell line KH39. Microcell hybrid lines were selected and propagated *in vitro *with 400 μg/ml Hygromycin B (Sigma, St. Louis, MO). MCHs, designated #3/KH39 (YYK2) and Δ3/KH39 (YYK1) were obtained from different fusion events [8].

Geneticin resistance gene tagged chr3 from MCH 903.1 was transferred into the poorly differentiated NPC cell line Hone 1. MCHs were selected and propagated *in vitro *with 400 μg/ml Geneticin (G418) (Sigma, St. Louis, MO) [7]. MCH #3/Hone1 (MCH4.8) was used for the present study.

Six-week old SCID mice were used for inoculations (10^6 ^cells per site). Mice were observed for tumor formation once a week up to 20 weeks. The tumors were excised under sterile conditions, explanted and expanded.

### 2. Fluorescence in Situ Hybridization (FISH)

The slides for FISH were prepared using standard techniques from the cells treated with colcemide, hypotonic solution and fixed in methanol: acetic acid (3:1).

DNA from CER1 specific PAC RP6-123i13 clone [11] was isolated using Qiagen columns (QIAGEN, Inc., Hilden, Germany) and labeled with biotin-dUTP (BIO-Nick Translation Mix, Roche Molecular Biochemicals, Mannheim, Germany). Double color FISH was performed as described [15] using chr3 specific painting probe labeled with FITC (Cambio, Cambridge, UK), combined with biotin labeled RP6-123i13. The biotin-labeled probe hybridization was detected with Cy3 conjugated streptavidin (Amersham Biosciences, GE Healthcare Worldwide). For analysis of Hone1 derived cells, we applied a second round of FISH using chr2 and chr12 specific painting probes labeled with Cy3 and biotin (Cambio, Cambridge, UK). The biotin-labeled probe hybridization was detected with Cy5 conjugated streptavidin (Amersham Biosciences, GE Healthcare Worldwide).

**Figure 2:**
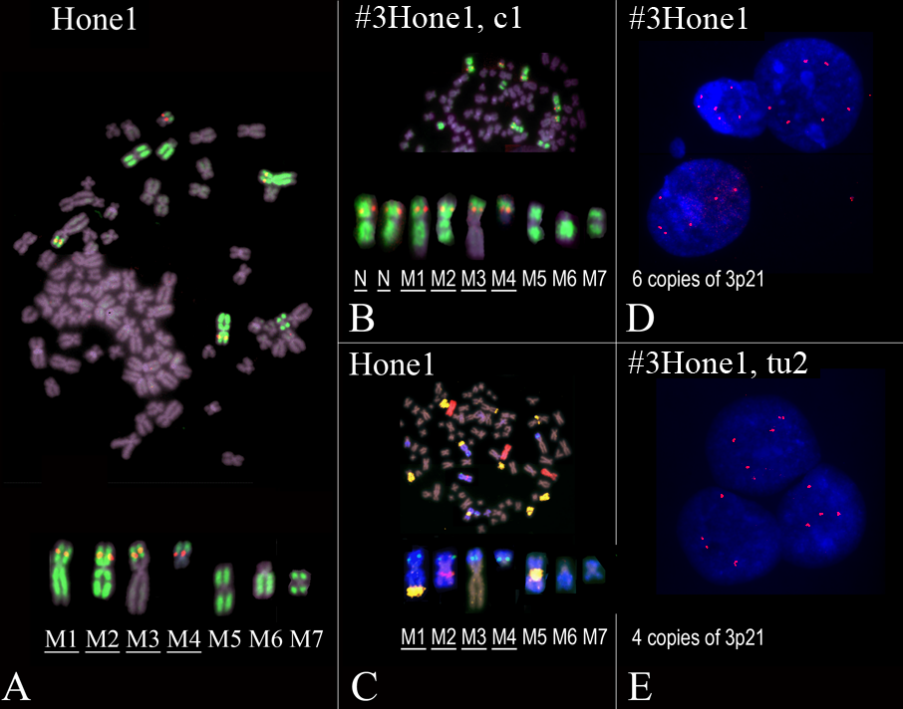
**FISH analysis of Hone1 derived cells**. **A**. Dual-color metaphase FISH (see METHODS) shows that in Hone1, parts of chr3 detected by chr3 painting probe (green) are included into different marker chromosomes (M1, M2 ...). The four underlined markers contain 3p21 as determined using the RP6-123I13 probe (red). **B**. After chromosome transfer, two additional normal chrs3 (N) are detected in #3/HONE1, clone 1. **C. **Identification of the marker chromosomes by four-probe metaphase FISH (see METHODS). Chr2, 3 and 12 painted parts are shown in red, blue and yellow, respectively. 3p21 RP6-123I13 signal is green. M1 has a translocation of a part of chr12. M2 carries a chr2 insertion. In M5, chr3 parts are translocated to a chr12 derived segment. **D. **Interphase FISH shows six RP6-123I13 probe signals (red) in the majority of the #3/Hone1 nuclei (blue DAPI staining). The sample was taken at the zero time point of *in vitro *propagation. This implies that the sample carries two supernumerary copies of the 3p21 (CER1) region, compared to four copies characteristic for Hone1. **E. **Interphase FISH, using the same probe, shows that #3/Hone1 cells have lost two supernumerary 3p21 copies after *in vivo *growth (tumor 2).

A fluorescence microscope (Leitz-DMRB, Leica, Heidelberg, Germany) equipped with a Hamamatsu C 4800 cooled CCD camera (Hamamatsu, Herrsching, Germany) was used to analyze minimum 20 metaphases and minimum 100 nuclei in each sample. For four-probe FISH, we captured the same metaphases after first and after second FISH round. Using Adobe Photoshop 7.0 (Adobe Systems, San Jose, Calif., USA) we superimposed the two images attributing to different probes different artificial colors (see figure 2C).

### 3. Multipoint FISH (mpFISH) for analysis of lost versus retained chromosomal fragments

DNAs prepared from chr3 specific plates (2 × 96-deepwell blocks) of Human FISH confirmed clones collection were obtained from BACPAC Resources Center (BPRC), CHILDREN'S HOSPITAL OAKLAND, Oakland, USA. All the probes or part of them were used in 2-color FISH to analyze chromosomes in Hone1 and in KH39 based MCHs. The probes were labeled either with biotin-dUTP or with digoxigenin-dUTP (BIO-Nick Translation Mix or Digoxigenin-Nick Translation Mix, Roche Molecular Biochemicals, Mannheim, Germany). The biotin-labeled probe hybridization was detected with Cy3 conjugated streptavidin (Amersham Biosciences, GE Healthcare Worldwide), the digoxigenin-labeled probe hybridization – using FITC conjugated anti-digoxigenin antibodies (Roche Molecular Biochemicals Mannheim). The presence or absence of signals on each marker chromosome was analyzed with a fluorescence microscope equipped with a Hamamatsu C 4800 cooled CCD camera (see details in [16]).

### 4. Analysis of polymorphic microsatellite markers

Eight polymorphic microsatellite markers within the 3p14.3-21.2, spanning 51–56 Mb, were used to score LOH: D3S1573, D3S3947, D3S3139, D3S1588, D3S1289, D3S1613, D3S1606, and D3S3721 [17]. Microsatellite analysis was performed by fluorescent PCR-based analysis on an Applied Biosystems Prism™ 3100 Genetic Analyzer (Applied Biosystems, Foster City, CA, USA). The PCR reactions were carried out in 10 μl reaction mix containing 10 × PCR Buffer II, 250 μM dNTP, 2.5 mM MgCl_2_, 10 pmol of each primer, 0.5 U of AmpliTaq Gold DNA Polymerase (Applied Biosystems, Foster City, CA, USA), 4 μM F-dCTP (for non-fluorescent labeled primers, D3S1289 and D3S1606 only) and sterile deionized water. 100 ng genomic DNA was amplified for 30 cycles at 94°C 30 sec, 55°C 30 sec, and 72°C 30 sec on a GeneAmp^® ^9700 PCR system (Applied Biosystems, Foster City, CA, USA). PCR products were then injected into semi-automated ABI Prism™ 3100 Genetic Analyzer for capillary electrophoresis and analyzed using GeneScan and GenoTyper 2.1 software.

## Results

### 1. Detection and analysis of clones by metaphase Fluorescence in Situ Hybridization (FISH)

To examine chromosome changes according to the scheme in figure 1, we have chosen KH39 and Hone1 derived MCHs that contained 2 extra chr3 copies with intact CER1 and FER regions. We designated them as **#3/KH39 **and **#3/Hone1. **They were previously named YYK2 [8] and MCH4.8 [7], respectively. As controls, we used the KH39 and Hone1 cell lines and the KH39 derived MCH that carried one exogenous chr3 with CER1/FER deletions. The latter is called Δ**3/KH39 **and was previously named YYK1 [8]. These MCHs were analyzed using dual-color metaphase FISH with chr3 painting and 3p21 CER1 specific RP6-123i13 probes. For each metaphase we determined a "**chr3 constitution" **based on the combination of normal **(N) **and abnormal **(marker, M1, M2...) **chrs3 (figure 2A and 2B). The chromosomes were distinguished by their morphology and by the presence (**underlined, N, M1, M2...) **or absence (M5, M6 ...) of the 3p21 RP6-123I13 signal. Dual-color FISH identified 7 markers in Hone1 derived cells, whereas the detection of the 8^th ^marker was difficult due to the very small 3q part (4Mb, according to our Multipoint FISH data). It was also difficult to distinguish between chromosomes N, M1, M2 and M5 (figure 2B). In order to define the chr3 constitution of #3/Hone1 unambiguously, we excluded the 8^th ^marker from analysis and identified the other markers by four-probe FISH (figure 2C).

**Figure 3:**
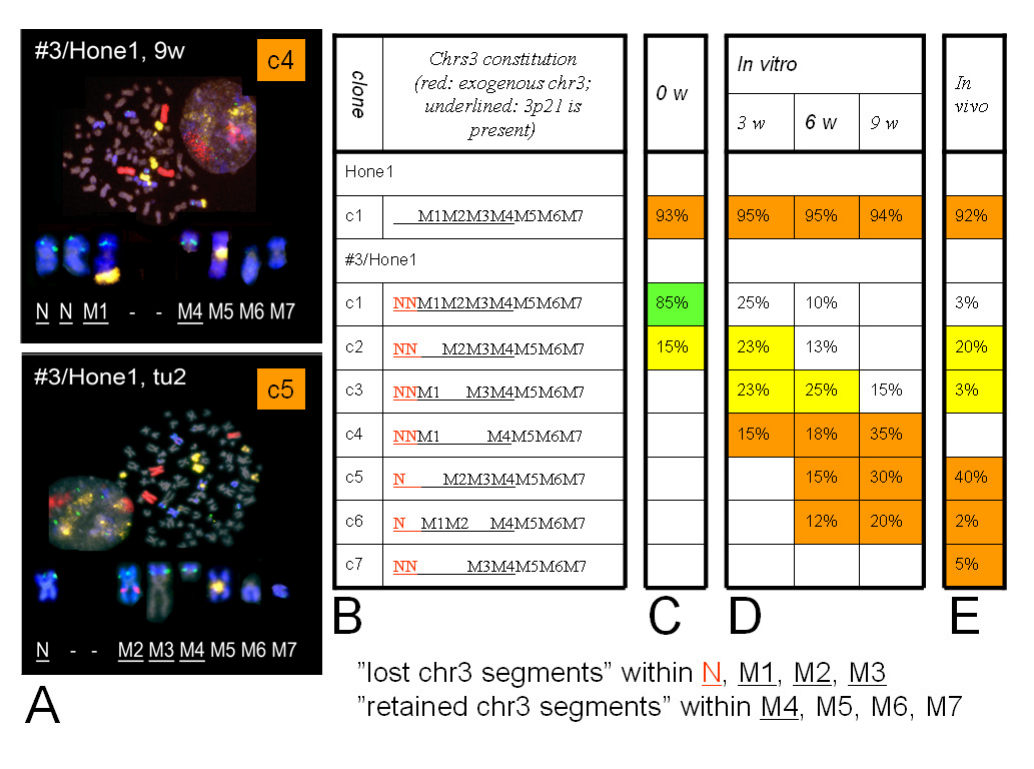
**Clonal changes in Hone1 and derived MCH #3/Hone1**. **A. **Four-probe FISH determines the chr3 constitution (see explanation to figure 2C) for #3/Hone1 samples taken at 9 weeks of *in vitro *propagation (c4) and in SCID tumor 2 (c5). **B. **Chr3 constitution for each clone. N- normal chr3; red specifies the exogenous chromosome; black are endogenous Hone1 derived markers, M1, M2 ... M7. Underlined: RP6-123I13 signal is present. **C-E. **Percentages of metaphases representing a particular clone at 0, 3, 6 and 9 weeks of propagation and in all SCID tumors analyzed. Orange, yellow and green shading: expanded clones with no, 1 and 2 supernumerary 3p21 copies, respectively, compared to Hone1. The term **"**expanded clone**" **refers to a clone that appeared at the given time or its representation was not decreased (ratio >0.8) compared to the previous time point. No shading: decreased representation from previous time point. Empty: clone is not present in the sample. Note that chr3 fragments, which were carried by N, M1, M2 and M3, were lost in more than one clone expanded during growth ("lost chr3 segments"), whereas ones included into M4, M5, M6 and M7 were never lost ("retained chr3 segments").

**Figure 4:**
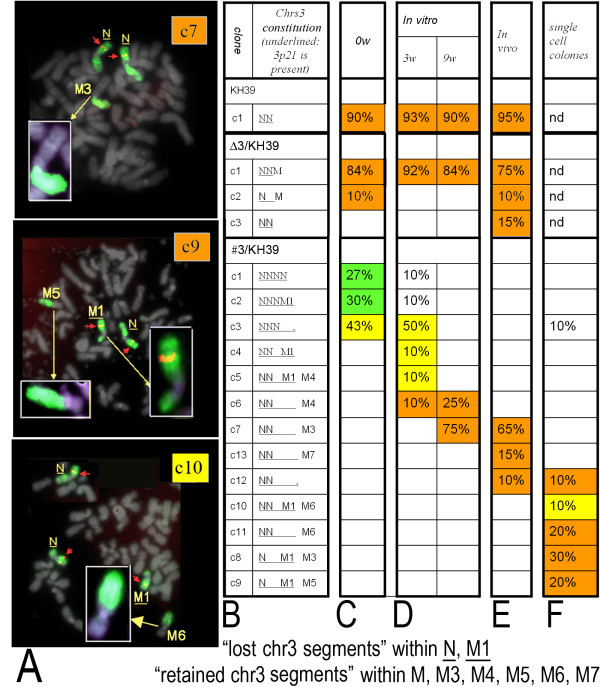
**Clonal changes in KH39, Δ3/KH39 and #3/KH39**. **A. **Dual-color FISH defines chr3 constitution (see explanation to figure 2A and 2B) for c7 in the #3/KH39, 9 weeks of *in vitro *propagation, and for c9 and c10, representing single cell colonies of the #3/KH39. Marker chromosomes are shown magnified. Orange text box with clone number (c7, c9): clones without supernumerary 3p21 copy. These have two RP6-123I13 signals (red arrows) per metaphase. Yellow text box: clone (c10) with one supernumerary 3p21 copy. It shows three RP6-123I13 signals per metaphase. **B. **Chr3 constitution for each clone includes N (cytogenetically normal chr3) and M, M1, M2 ... M7 (different marker chromosomes). Underlined: the present or lost chromosome had 3p21 FISH signal. **C-F. **Percentages of metaphases representing a particular clone at 0, 3 and 9 weeks of propagation; in all analyzed SCID tumors and in 10 single cell colonies (not done for KH39 and Δ3/KH39, "nd"). The tumors that derived from #3/KH39 and Δ3/KH39 have been analyzed previously [8]. Orange, yellow and green shading: expanded clones with no, 1 and 2 supernumerary 3p21 copies, respectively, compared to KH39. The term **"**expanded clone**" **refers to a clone that appeared at the given time or its representation was not decreased (ratio >0.8) compared to the previous time point. No shading: decreased representation compared to previous time point. Empty: clone is not present in the sample. Note that chr3 fragments, which were carried by N and M1, were lost in more than one clone expanded during growth ("lost chr3 segments"), whereas ones included into M, M3, M4, M5, M6 and M7 were retained as extra chr3 copies in the expanded clones ("retained chr3 segments").

Whenever a given chr3 constitution was found in more than 10% of the analyzed metaphases in a cell sample, we regarded these metaphases as belonging to the same cell **clone (c1, c2 ...)**. The clonal "**representation" **(% of metaphases with a particular chr3 constitution) was determined in samples taken at 0, 3, 6 and 9 weeks of *in vitro *propagation and in Severe Combined Immunodeficiency (SCID) mice derived tumors (figures 3 and 4). The term **"expanded clone" **refers to a clone that appeared at the given time or its representation was not decreased (ratio >0.8) compared to the previous time point. The expanded clones were classified according to the number of 3p21 RP6-123i13 FISH signals per metaphase. This was 4 in Hone1 and 2 in KH39. Expanded clones with 2, 3 or 4 RP6-123i13 FISH signals in KH39 derived cells and 4, 5 or 6 signals in Hone1 derived cells, were classified as clone without, with 1 or 2 **supernumerary 3p21 copy numbers, **respectively (see orange, yellow or green colors in figures 3 and 4).

### 2. Clonal changes in #3/Hone1 and #3/KH39

**Figure 5:**
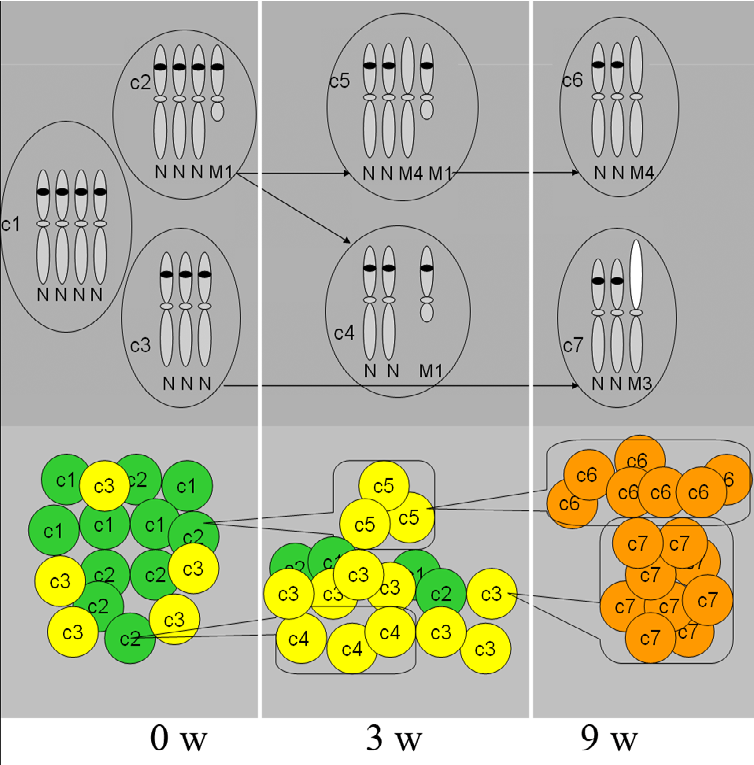
**Schematic illustration of dual-phase clonal expansion of cells that lose first one (yellow) then the second (orange) supernumerary 3p21 copy in #3/KH39 MCH propagated *in vitro *during 0, 3 and 9 weeks (0w, 3w and 9w, respectively)**. Chr3 constitution and derivation of each clone is shown at the top. 3p21 is indicated as a black band on 3p. In comparison to KH39, which has 2 copies of this region, 4, 3 and 2 copies in a clone correspond to 2, 1 and no supernumerary copies, respectively. The cells representing such clones are shown below in green, yellow and orange colors, respectively. Clone c5, derived from c2 by a chr3 rearrangement (M4), gave rise to c6 by loss of M1. Loss of one normal chr3 in c2 led to expansion of c4. Translocation of the long arm of chromosome 1 (white on M3) to 3q in c3 produced clone c7, which expanded as the largest clone.

**Figure 6:**
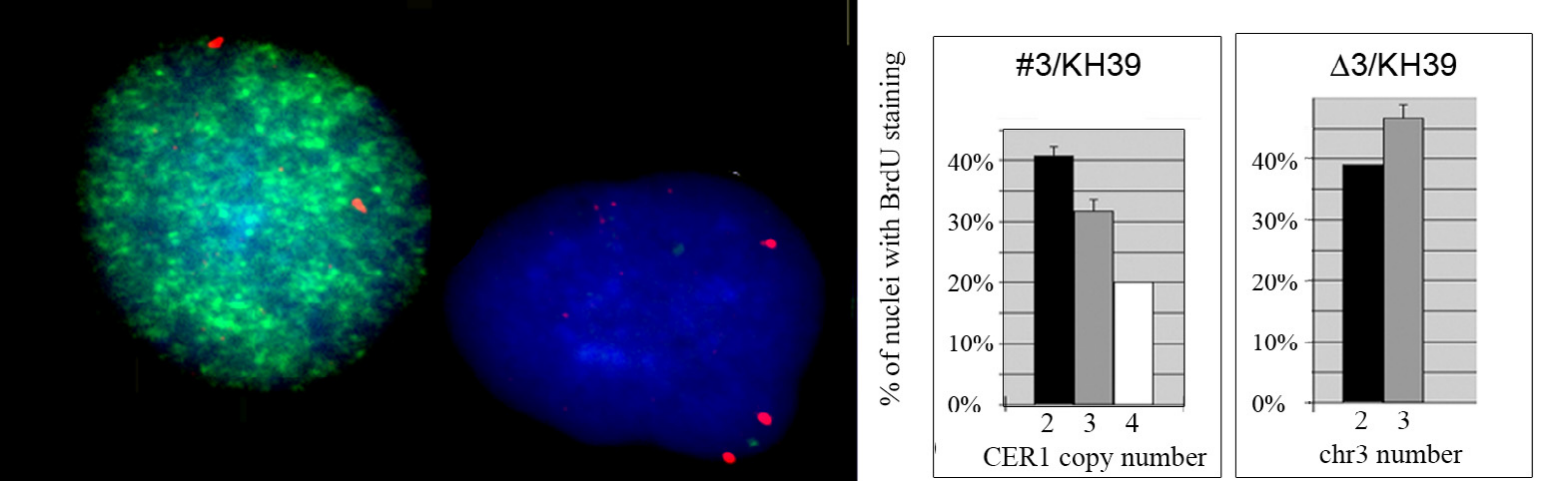
**The S-phase fraction of cells increases with loss of an extra 3p21 copy**. Living cells of the MCHs #3/KH3 and Δ3/KH39 were incubated with BrdU in final concentration 30 μg/ml during 1 hour followed by fixation with methanol: acetic acid (3:1). FISH with biotin-labeled 3p21-specific RP6-123i13 probe and centromere-specific probe was performed to analyze the #3/KH3 and the Δ3/KH39 respectively. The probe signal was developed using Cy3-conjugated streptavidin **(red), **while FITC-conjugated anti BrdU antibodies (Roche Molecular Biochemicals Mannheim, Germany) identified cells in S-phase **(green). **Percentage of S-phase cells in subpopulations of cells with different 3p21 copy numbers in the #3/KH39 and with different chr3 copy number (counted by number of centromeres) in the Δ3/KH39 was calculated in more than 100 nuclei. The calculation was repeated three times when number of cells allowed. The average values and standard deviations are shown.

Figures 3 and 4 show changes in clonal representation in Hone1 and KH39 derived cells observed during a period of 9 weeks of *in vitro *propagation and after *in vivo *growth. The control KH39 and Hone1 did not show any cytogenetic changes. The MCH Δ3/KH39 that had an extra chr3 copy with 3p21 deletions retained its dominant clone during 9 weeks of *in vitro *propagation and following *in vivo *growth. In contrast, the MCHs #3/KH39 and #3/Hone1, which had extra copies of chr3 with intact CER1 and FER, exhibited notable changes. After 3 weeks of cultivation, the clones with 1 supernumerary 3p21 copy (figure 3D and 4D, yellow) replaced the clones with 2 supernumerary 3p21, which were dominant at week 0 (figure 3C and 4C, green). After 9 weeks, clones that have lost all supernumerary 3p21 copies, took over (figure 3D and 4D, orange). This two-step clonal expansion is in line with the "mandatory chromosomal segment copy number" hypothesis (see figure 1B). Analysis of the chr3 constitution of different clones that emerged during MCH propagation shows that they are probably derivatives of the initial clones. Having growth advantage, they expanded during propagation. A hypothetical chain of changes with emergence and expansion of derivative clones is shown for #3/KH39 in figure 5. The growth advantage of cells with one supernumerary copy of 3p21 compared to two and with no supernumerary copies compared to one copy was confirmed directly by Bromodeoxyuridine (BrdU) incorporation into the KH39 derived MCHs (figure 6).

Clones with one or no supernumerary 3p21 copies expanded in the *in vivo *derived #3/KH39 and #3/Hone1 tumors as well (see figures 3E and 4E). Another indication of the growth and colony formation promoting effect of an extra-3p loss was obtained from the single cell colony analysis of #3/KH39 (figure 4F). Ten largest #3/KH39 single cell colonies were picked up and analyzed. Out of these 10 subclones, 8 did not have supernumerary 3p21 copy. Only 2 had one supernumerary 3p21 copy. No clones were found with two supernumerary 3p21 copies, in spite of their overrepresentation in the MCH harvested to obtain colonies (#3/KH39, time point zero of *in vitro *propagation, figure 4C).

Interphase FISH with the RP6-123I13 probe (see figures 2D and 2E) confirmed the results of chromosome analysis. Hone1 and KH39 gave 2 and 4 RP6-123I13 signals, respectively, in the majority of the nuclei and this proportion did not change during *in vitro *and *in vivo *growth. At the beginning, the prevalent cell population in #3/Hone1 and #3/KH39 contained 6 and 4 copies, respectively, of 3p21. After *in vitro *propagation of #3/KH39 and #3/Hone1 during 3 and 6 weeks, respectively, one supernumerary 3p21 copy was lost in the majority of cells. After 9 weeks *in vitro *propagation, the major subpopulation in both MCHs did not contain any supernumerary 3p21 copy. The same was found after *in vivo *growth in all #3/KH39 derived tumors and in 3/5 #3/Hone1 derived tumors.

### 3. Rigidity of the initial copy number

**Figure 7:**
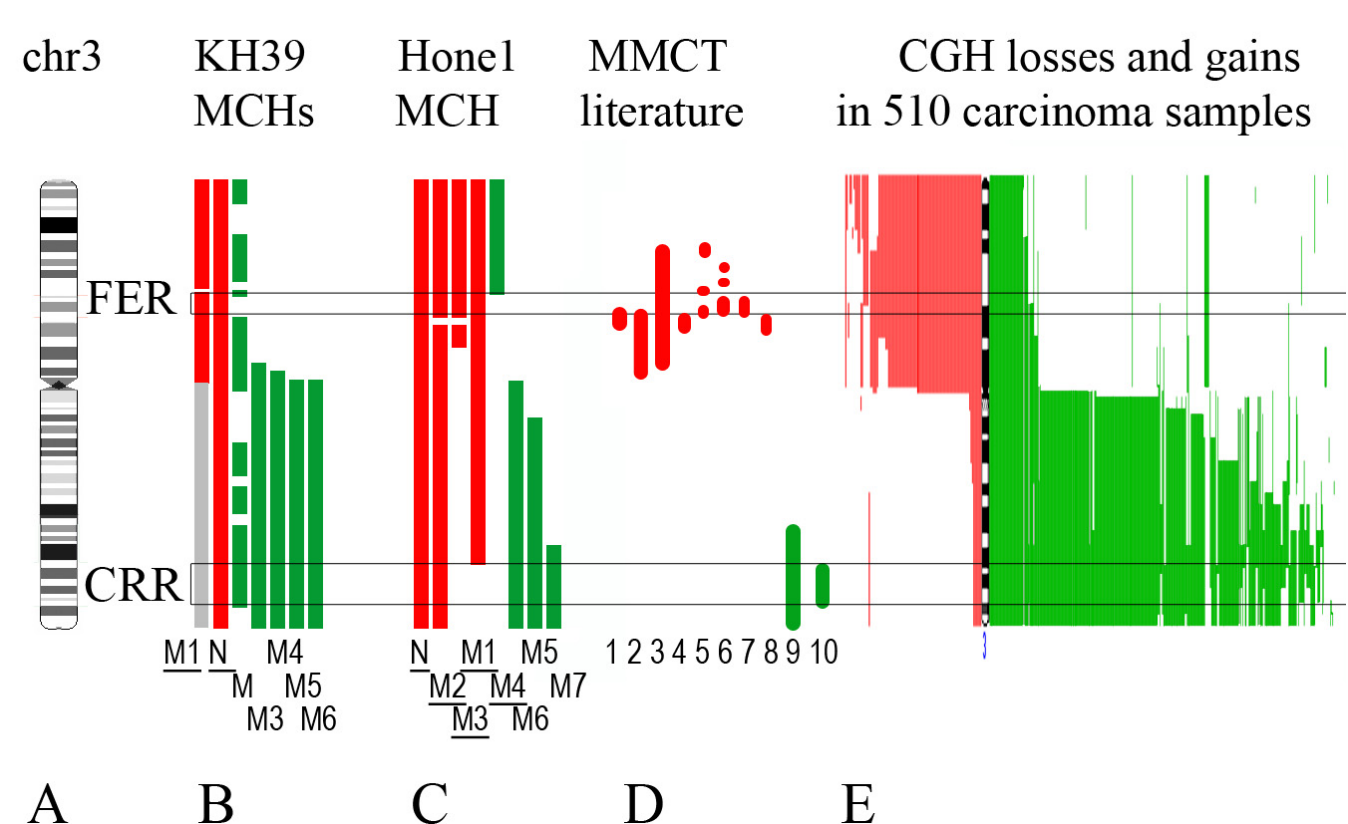
**Variation in the rigidity of initial copy number in different chr3 segments**. **A. **Chr3 ideogram with the localization of FER and CRR regions (empty horizontal bars through the figure). **B-C. **Analysis of the genetic material of the expanded clones shows that elimination of the "lost chr3 segments" in contrast to the "retained chr3 segments" was associated with growth advantage (see figures 3 and 4). Therefore we compare these "lost chr3 segments" (red vertical bars) with the "retained chr3 segments" (green vertical bars) in order to find a "mandatory copy number region. MpFISH identified chr3 regions that are present on the "lost" (red) and the "retained segments" (green) in KH39 and Hone1 derived MCHs (see figures 3 and 4) (gray: the region was not analyzed). **D. **Summary of data concerning the regions involved in growth suppression following chr3 transfer into tumor cell lines, as reported at the time of publication. Note that many of the early MMCT studies were limited by availability of genetic markers for assaying content of hybrids or were not performed comprehensively to include genetic marker analysis of both chromosome arms. **Red: **reported regions associated with the growth suppression: (1) in ovarian carcinoma line HEY [19]. (2) in breast carcinoma line 21NT [22]; (3) in RCC cell line RCC23 [20]; (4) in RCC cell line KC12 [21]; (5) and (6) in mouse fibrosarcoma A9 cell line [18] and [9]; (7) in NPC Hone1 cell line [7]; (8) in esophageal squamous cell carcinoma cell line SLMT-1 [23]. **Green: **common retained region (CRR) tends to persist in mouse fibrosarcoma A9/human chr3 MCHs after 4 consecutive passages through SCID mice. (9) [24]; (10) the region was reduced to the segment between PCR markers D3S1282 (3q26.2) and D3S1262 (3q27.3) (our unpublished data). **E. **Data about losses and gains of chr3 segments in 510 cases of colorectal, renal, parathyroid, lung, pancreas, breast, cervical, fallopian tube, ovarian, vaginal and tonsil carcinomas reported in NCBI SKY/M-FISH and CGH Database [25]. Each red line corresponds to the loss, each green – to the gain of the segment in a particular tumor sample.

Analyzing changes of chr3 constitution during MCHs *in vitro *and *in vivo *growth, we have found that chromosomes, which contained 3p21, were preferentially lost compared to the chromosomes that did not have it (see underlined versus not underlined chromosomes in Figures 3B and 4B). This suggests that the rigidity of the initial copy number is different for different chromosomal segments. To examine the tolerance of different chr3 regions to copy number change, we performed Multipoint FISH (mpFISH) analysis (see METHODS) on the marker chromosomes. Chromosomes N and M1 were repeatedly lost in the #3/KH39 derived expanded clones; in the #3/Hone1 these were N, M1- M3**("lost chr3 segments") **(see figures 3 and 4). Markers that were never lost and fragments retained as an extra-copy after *in vitro *propagation and *in vivo *growth, were M in Δ3/KH39; M3-M7 in #3/KH39 and M4, M5-M7 in #3/Hone1 **("retained chr3 segments"). **The results of mpFISH analysis of the "lost" versus the "retained chr3 segments" are shown in figure 7B and 7C (red versus green bars).

The previously reported frequently eliminated region (FER) at 3p14.3-p21.2 was part of all "lost", but none of the "retained chr3 segments". This region is most resistant to copy number gain. In contrast, 3q26.3-q27 was present preferentially in the "retained chr3 segments" being thus most tolerant to copy number gain.

To relate our results to previous findings, we looked for published data concerning chr3 transfer into tumor cells [7,9,18-23]. The search showed that 3p14.3-p21.2 was frequently involved in growth suppression (red in Figure 7D), while 3q26.3-q27 was regularly retained after *in vivo *growth of mouse fibrosarcoma/human chr3 MCHs ([2,24] and our unpublished data). This region was designated CRR (Common Retained Region) (green in Figure 7D).

We have also used CGH Case Comparison Tool [25] to analyze FER and CRR involvement in 510 cases of colorectal, renal, parathyroid, lung, pancreas, breast, cervical, fallopian tube, ovarian, vaginal and tonsil carcinomas (figure 7E). This search showed that 208 cases had 3q gains with highest incidence within the CRR. The entire 3p14-p21 region or part of it was lost in 84 cases, 164 cases showed gains on chr3 excluding this region.

### 4. The consistently lost 3p (FER) region can be of either exogenous ("normal") or endogenous ("tumor") origin

**Figure 8:**
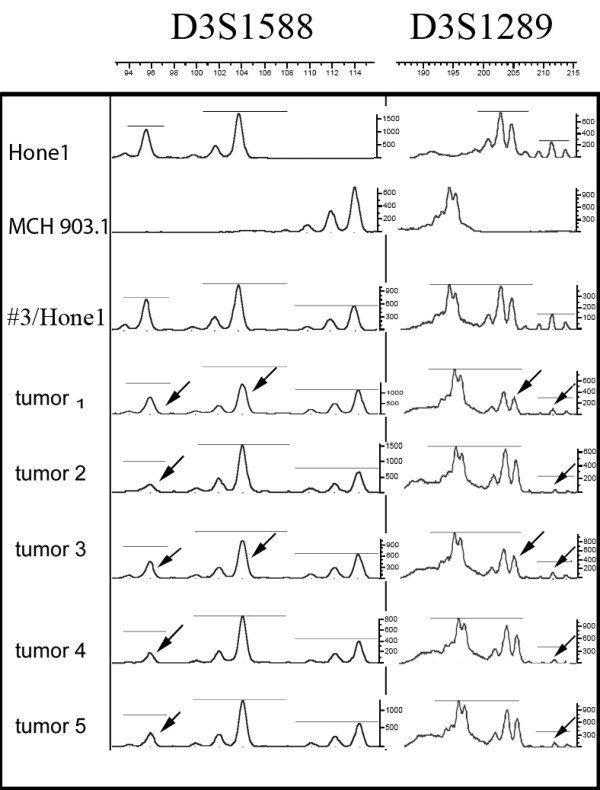
**Polymorphic microsatellite marker analysis of Hone1, #3/Hone1 and 5 derived tumors**. PCR products of genomic DNA amplification using 2 polymorphic microsatellite markers D3S1588 and D3S1289, located within 3p21.1 (54.1 Mb) and 3p14.3 (54.5 Mb), respectively, were analyzed using semi-automated ABI Prism™ 3100 Genetic Analyzer for capillary electrophoresis. The results are presented in graphical view according to GeneScan and GenoTyper 2.1 software. Recipient Hone1, donor MCH903.1, hybrid #3/Hone1 and 5 tumors derived from the #3/Hone1 were genotyped. Horizontal lines above the peaks show the original ratio between peak heights (characteristic for the #3/Hone1). Arrows indicate relative reduction of endogenous (derived from Hone1) peaks in the tumor compared to the #3/Hone1 used for SCID mice inoculations. This reduction was detected for both markers in two independent experiments.

Figure 3B shows that the exogenous (red) and the endogenous (black) chromosomes could be lost alternatively during *in vitro *and *in vivo *propagation of #3/Hone1. Eight polymorphic microsatellite markers within and close to FER were included in the LOH study (figure 8). Hone1 was heterozygous for 6 of 8 markers, suggesting that the endogenous marker chromosomes contain two alleles for this region. Further analysis of chr3 donor MCH903.1 and hybrid #3/Hone1 has proven that both recipient and donor alleles were present in #3/Hone1. In order to examine the losses in the tumors we selected two markers, D3S1588 and D3S1289, with three clearly distinguishable peaks corresponding to endogenous and exogenous alleles. Analysis of 5 tumors did not show complete loss of any of the alleles. There were reproducible changes in the balance between peak sizes however, corresponding to the cytogenetical finding of the alternative loss of either the endo- or the exogenous chromosomes.

In #3/KH39 it was impossible to distinguish between exo- and endogenous chromosomes cytogenetically. On the basis of comparative cytogenetic and LOH tests we have reported earlier that the rearrangements could affect either the exo- or the endogenous chr3 in the #3/KH39 SCID tumors [8]. In the present study, we found indirect evidence that the endogenous chr3 could be rearranged or lost in single cell derived colonies. Figure 4B shows that c8 and c9 retained only one N, indicating that at least one endogenous chr3 was lost or rearranged; c12, had the chr3 constitution NN and was growing in selective medium (see METHODS). This suggests that at least one of these chrs3 was exogenous.

The equivalence of endo- and exogenous losses gives strong support to the idea that the copy number of certain chr3 segment is strictly controlled and brought back to its original value by selection, going after chr3 transfer (see figure 1B).

## Discussion

We have previously shown that the transfer of a normal chr3 into an RCC and a NPC tumor cell lines suppressed tumorigenicity [7,8]. *In vitro*/*in vivo *growth of the derived intact chr3 carrying MCHs was associated with chr3 losses and rearrangements. Cell clones that have lost either an exogenous or an endogenous supernumerary 3p expanded preferentially. Due to the loss of certain chr3 segments, the original 3p copy number that existed in the tumor cell prior to the transfer of normal chromosome was re-established. This indicates that these aneuploid tumor cells are dependent on a precise balance that they have evolved with regard to some chromosomal segments, but not others. The differential sensitivity of chr3 segments to the change of the numerical equilibrium, seen both in the nearly diploid KH39 and the highly rearranged Hone1 line, is especially interesting (see Figure 7). The copy number of the FER (3p14.3-p21.2) region was guarded with particular rigor in both. In contrast, an extra 3q was often retained, even after long propagation. The copy number of 3q26.3-q27 (CRR) was higher than the original recipient cell line number in all 9 analyzed *in vitro *growth selected clones and in 7 of 8 *in vivo *selected clones.

The copy number of FER was two in the hyper-diploid KH39 and 3 in the hypo-tetraploid Hone1 line. Chr3 transfer increased the FER copy number by 2 after chr3 transfer in both cell lines changing this region status from "loss" (copy number less than ploidy level) to "gain" (copy number higher than ploidy level). Extra FER copies were eliminated during *in vitro *propagation and *in vivo *passage from both MCHs although at a different rate. Even after long propagation, one additional 3p copy was detected in a fraction of cells in the #3/Hone1 (see Figure 3), but not in the #3/KH39 (see Figure 4). This is consistent with the "lower gain" of FER by one copy number increase in #3/Hone1 (4 copies in hypo-tetraploid cell line) compared to #3/KH39 (3 copies in hyper-diploid cell line).

Several studies show that introduction of exogenous DNA into cultured tumour cells leads to chromosomal instability, disturbing the gene equilibrium [26]. An alternative hypothesis for restoration of tumourigenicity could then involve other mechanisms, than loss of 3p, like gain or loss of other chromosomes. Multiplex FISH (M-FISH) analysis of #3/KH39 MCHs showed, that other chromosomes were rearranged during the propagation of the MCHs as well [6]. The involved chromosomes were regularly polysomic (present originally in more than 2 copies) and served as partners for unbalanced chr3 translocation. This led us to conclude that polysomy is associated with structural chromosome instability [6]. The recurrent involvement of chr3 and the differential involvement of 3p and 3q were obvious in our previous and present studies. This selectivity of the loss indicates that both, the induction of instability by MMCT and the selection for specific 3p/3q balance, take place in the MCHs.

Several tumor growth inhibiting genes are known to reside on 3p [1,2]. This may explain the tendency of the entire 3p to equilibrate at a low copy number (see Figure 7). The known TSG, *FHIT *(fragile histidine triad) at 3p14 [27] centromeric to FER, was only found in one copy in the hypo-tetraploid Hone1. Interstitial deletions within this gene were detected on two marker chromosomes M2 and M3 in Hone1. After *in vitro *propagation of #3/Hone1, both M2 and M3 were eliminated with the same frequency as the normal chrs3 and M1, which had an intact *FHIT *(c3, c4, c5 and c6 in Figure 3). However, after *in vivo *growth, the normal chr3 and M1 were lost more frequently than the other markers (c2 and c5 represent 20% and 40% of *in vivo *tumor cells, respectively, in Figure 3). *FHIT *mRNA was expressed in KH39 and in the derived hybrids whereas the protein level was slightly decreased after *in vivo *growth (our unpublished data). This suggests that the dependence of the tumor on a correct FER copy number is stronger than what can be attributed to *FHIT*. Still the *FHIT *copy number may have an additional impact *in vivo*. Telomeric to FER a lung cancer TSG region (LUCA) was reported to contain multiple potential TSGs. This region is frequently affected by deletions in lung and other epithelial cancers. Several genes located here showed reduced mRNA levels in lung cancer cell lines and for some of these additional functional data supported their role as TSGs. None of the candidate TSGs (*CACNA2D2*, *PL6*, *101F6*, *NPRL2*, *BLU*, *RASSF1*, *FUS1*, *HYAL2, HYAL1 *and *SEMA3B*) showed a frequent mutation rate in lung cancer samples however, suggesting that they may function in dose-dependent manner (see [1] for review). Earlier we have examined the expression of 34 human 3p-genes from known cancer-related regions including LUCA in mouse fibrosarcoma A9 based MCHs and derived tumors. We have found the transcript of the solute carrier family 38 member 3 gene (*SLC38A3*) was not found in 8 tumors and was greatly reduced in 1 out of these 9 tumors. Transcription of this gene was also impaired in 5 RCC cell lines analyzed [28]. However, the LUCA region does not overlap our "mandatory copy number region". It was present in supernumerary copes in Δ3/KH39, which grew without any losses. The genes from this region did not change their expression in the KH39 derived MCHs (our unpublished data). In #3/Hone1 the region was present on M4 marker chromosome, which was never lost in the expanded clones (see figure 3). In summary, there are probably several genes on 3p, which are preferred at low copy numbers in a tumor. As consequence, the tumor is dependent on its gene copy number balance, but for some genes the quantitative rigidity is stronger than for others. FER is an example of a "mandatory copy number region".

The tolerance of CRR to copy number gain may be due to the presence of growth promoting genes. It was preferentially retained in our earlier studies on a mouse tumor MCH system [9]. Gains on 3q are frequently detected in human tumors. NCBI SKY/M-FISH and CGH Database analysis [25] shows that in different carcinomas the frequency of gains affected 3q26 and 3p21-pter was highest and lowest, respectively (see figure 7D). Interestingly, no 3p21 gain was found in 98 cases of such carcinoma types as fallopian tube, kidney, parathyroid, lung and breast carcinomas. This was the only autosomal region that retained the same "no gain" copy number in all 98 cases.

When we analyzed 10 RCC cell lines, no FER gain was found in spite of centromere gain in 5 and 3q26-qter gain in 8 of the 10 cell lines (data not shown). Seven of these had FER deletions, but only three of the seven cell lines showed LOH for polymorphic microsatellite markers in FER [see Additional file 1]. This suggests that 3p deletions may occur preferentially when chr3 is polysomic (more than 2 copies) and could act by copy number reduction.

Taken together, these findings affirm the notion that the copy number restriction at specific chromosomal segments, exemplified by 3p14-p21, is critically important for tumor growth. In contrast, copy number gain in 3q26-q27 is positively associated with tumor growth. This apparent disequilibrium can be readily achieved in aneuploid cells, e.g. by the structural instability of polysomic chromosomes, with breakage within late replicating regions of supernumerary chr3, often close to the centromere [6]. Translocations of 3q fragments would result in the loss of supernumerary 3p and retention of 3q, generating the optimal balance of chr3 segment copy numbers for the given tumor line.

## Conclusion

Short arm of human chr3 shows frequent losses in human carcinomas, in contrast to long arm that is affected by gains and amplifications. The regions 3p21 and 3q26 are often involved in those reciprocal changes. Our earlier studies with MCHs, generated by the introduction of a normal human chr3 into murine or human tumor cells have already led to the notion, that 3p may contain tumor growth antagonizing and 3q – growth promoting genes. Following tumor growth in SCID mice, MCHs regularly lost defined regions in the 3p14-p21 area, designated as CER1 and FER. In contrast, 3q26-qter (CRR) was maintained even after serial mouse tumor passages.

In this paper, we showed that the *in vitro *and *in vivo *losses affecting the 3p14.3-p21.2 (FER) segment in tumor cells with an introduced exogenous normal human chr3 may alternatively target the exogenous or an endogenous chr3. This shows a dosage effect and speaks against any qualitative, e.g. mutational inequality between the tumor derived and the normal chromosome. The intolerance to this dosage change does not apply to 3q where the increased copy number is readily maintained and apparently favored.

## Competing interests

The author(s) declare that they have no competing interests.

## Authors' contributions

MK designed the study, carried out the cytogenetical studies, database searches and drafted the manuscript. ED carried out the cell culture, SCID mice experiments, participated in mpFISH experiments and has been actively involved in the result interpretations and drafting the manuscript. WLY carried out the LOH study. AS helped with cytogenetical studies. LF participated in cell culture and SCID experiments. YY produced KH39 based MCHs. IK participated in definition and restriction of FER and CRR borders. YC produced Hone1 based MCHs. MLL participated in the coordination, result interpretation and helped to draft the manuscript. ES have made substantial contribution to conception. GK and SI have made substantial contribution to conception and design of the study, to result interpretations and have been actively involved in the drafting the manuscript. All authors read and approved the final manuscript.

## Pre-publication history

The pre-publication history for this paper can be accessed here:



## Supplementary Material

Additional file 1 Chr3 alterations in RCC cell lines speak in favor of "Mandatory chromosomal segment copy number" model. Click here for file
