# Supplementary material for: Mandatory chromosomal segment balance in aneuploid tumor cells
Source: BMC Cancer. 2007 Jan 26;7:21. doi: 10.1186/1471-2407-7-21 (PMC1794251; doi:10.1186/1471-2407-7-21)
Supplement: Additional file 1 — Chr3 alterations in RCC cell lines speak in favor of "Mandatory chromosomal segment copy number" model. [file 1471-2407-7-21-S1.pdf]

Additional Material.

Chr3 alterations in RCC cell lines speak in favour of "Mandatory chromosomal segment copy number" model.

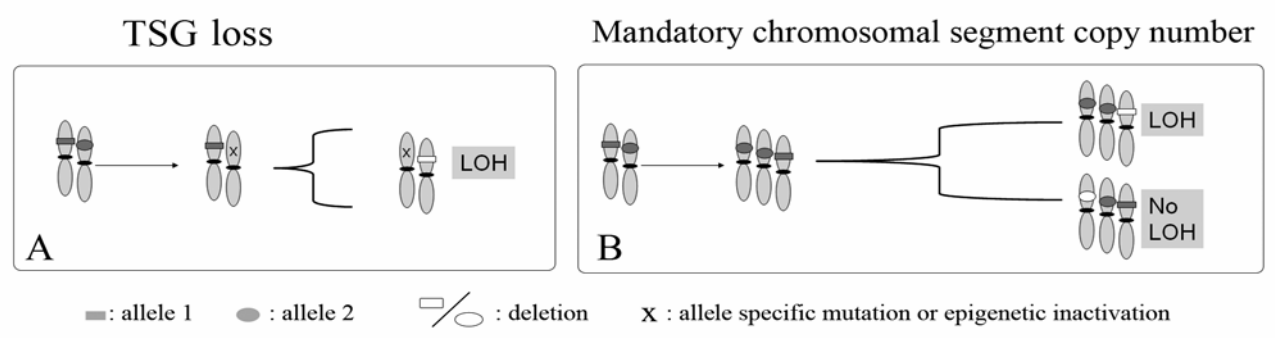

A. "TSG loss" model in human tumors would imply functional of physical elimination of both tumor suppressor alleles. Therefore deletion would be effective if it would eliminate one allele of the gene leading to LOH in majority of tumors.

B. In aneuploid human tumors the "Mandatory chromosomal segment copy number" model considers that a tumor suppressor gene copy number gain having chance to occur with numerical chr3 changes would have to be diminished by deletion of the 3p14-p21 region, not necessarily accompanied by LOH and tendency to keep 3q gain.

In order to test applicability of these models, we have studied FER status in several RCC cell lines.

Preliminarily we performed metaphase chromosome analysis using FISH with chr3 centromere specific probe and 10 FER specific probes. Based on this analysis we have selected 7 cell lines that had FER deletions. We considered the FER region deleted, when derivative chr3 (rearranged chromosome that has centromere from chr3) did not have FER FISH signals and FER signals were not present on chr3 translocation to other chromosomes. We analysed these 7 cell lines for retention of heterozygosity using eight 3p14.3-p21.2 (FER) microsatellite markers (see table below). Four out of seven cell lines retained heterozygosity of several FER polymorphic markers. Taking in account that in all these lines the deletion spanned the entire FER (all FER FISH markers were absent from the derivative chromosomes) we concluded that the copy number loss of FER was not accompanied by LOH in these 4 cell lines. This supported the **"**Mandatory **c**hromosomal segment copy number" model.

RCC cell line	Ploidy, near	FISH results			FER genotyping. 1: one allele; 2: two alleles								
		centromere copy number	FER copy number	FER deleted der 3	Marker	D3S1573	D3S3947	D3S3139	D3S1588	D3S1289	D3S1613	D3S1606	D3S3721
					Position, Mb	51.1	51.4	53.9	54.1	54.4	54.9	55.6	55.9
A498	3	3	2	1		1	1	1	1	1	1	1	1
TK164	3	4	3	1		1	1	1	1	1	1	1	1
UOK123	2	3	2	1		Homozygosity for 3p14.2-p22, published*							
Caki 1	3	3	2	1		**2**	1	1	1	**2**	**2**	**2**	**2**
UOK115	3	4	2	2		**2**	1	1	1	**2**	**2**	1	**2**
UOK125	2	3	2	1		1	1	1	**2**	**2**	1	1	**2**
UOK147	4	5	4	1		1	1	1	**2**	**2**	1	1	**2**

Method. *FER FISH and genotyping analyses in 7 RCC cell lines A498, UOK-115, UOK-123, UOK-125, UOK-147, TK164, (Bear et al., 1987; Gnarra et al., 1994; Tomita et al., 1996; Yano et al., 1988)and Caki 1 (ATCC catalog No.HTB46). We have analysed minimum 20 metaphases using chr3 enumeration centromere specific probe to identify derivatives of chr3 and using FER-specific probes to detect FER deletions. FER-specific probes were obtained from Human FISH confirmed clones collection, BACPAC Resources Center (BPRC), CHILDREN'S HOSPITAL OAKLAND, Oakland, USA. We have chosen 12 probes located at 3p14.3-p21.3 between Mb positions 51 and 58. These were CTD-2199g5, RP11-122d19, RP11-124o2, RP11-189k9, RP11-89k24, RP11-39l1, RP11-169g24, RP11-120c2, RP11-229a12 and RP11-80h18. We counted also number of chromosomes in the dominant clone to estimated ploidy level.*

Genotyping with 8 FER polymorphic microsatellite markers (see supplementary figure 3) showed that 4 out of these 7 cell lines retained FER heterozygosity in spite of presence of the deletion.

*: genotyping was not done for this cell line in the present study, but we relied on our previously published data about mono-allelic status of 3p14.2-p22 in it (Alimov et al., 2000)

Reference:

Alimov A, Kost-Alimova M, Liu J, Li C, Bergerheim U, Imreh S, Klein G and Zabarovsky ER. (2000). *Oncogene*, **19,** 1392-9.

Bear A, Clayman RV, Elbers J, Limas C, Wang N, Stone K, Gebhard R, Prigge W and Palmer J. (1987). *Cancer Res*, **47,** 3856-62.

Gnarra JR, Tory K, Weng Y, Schmidt L, Wei MH, Li H, Latif F, Liu S, Chen F, Duh FM and et al. (1994). *Nat Genet*, **7,** 85-90.

Tomita Y, Bilim V, Kawasaki T, Takahashi K, Okan I, Magnusson KP and Wiman KG. (1996). *Int J Cancer*, **66,** 322-5.

Yano H, Maruiwa M, Sugihara S, Kojiro M, Noda S and Eto K. (1988). *In Vitro Cell Dev Biol*, **24,** 9-16.
